# Analysis of Wheat Virome in Korea Using Illumina and Oxford Nanopore Sequencing Platforms

**DOI:** 10.3390/plants12122374

**Published:** 2023-06-19

**Authors:** Hyo-Jeong Lee, Sang-Min Kim, Rae-Dong Jeong

**Affiliations:** 1 Department of Applied Biology, Institute of Environmentally Friendly Agriculture, Chonnam National University, Gwangju 61185, Republic of Korea; hjhjhj8@naver.com; 2 Crop Foundation Research Division, National Institute of Crop Science, Rural Development Administration, Wanju 55365, Republic of Korea; kimsangmin@korea.kr

**Keywords:** plant virus, wheat, virome, high-throughput sequencing, nanopore sequencing

## Abstract

Wheat (*Triticum aestivum* L.) is one of the most important staple crops in the world, along with maize and rice. More than 50 plant viruses are known to infect wheat worldwide. To date, there are no studies on the identification of viruses infecting wheat in Korea. Therefore, we investigated virome in wheat from three different geographical regions where wheat is mainly cultivated in Korea using Oxford Nanopore Technology (ONT) sequencing and Illumina sequencing. Five viral species, including those known to infect wheat, were identified using high-throughput sequencing strategies. Of these, barley virus G (BVG) and *Hordeum vulgare* endornavirus (HvEV) were consistently present in all libraries. Sugarcane yellow leaf virus (SCYLV) and wheat leaf yellowing-associated virus (WLYaV) were first identified in Korean wheat samples. The viruses identified by ONT and Illumina sequencing were compared using a heatmap. Though the ONT sequencing approach is less sensitive, the analysis results were similar to those of Illumina sequencing in our study. Both platforms served as reliable and powerful tools for detecting and identifying wheat viruses, achieving a balance between practicality and performance. The findings of this study will provide deeper insights into the wheat virosphere and further help improve disease management strategies.

## 1. Introduction

Wheat (*Triticum aestivum* L.) grows well even in dry and barren environments and does not require much labor, making it easier to cultivate widely. It contains various bioactive compounds, making it one of the most important cereal crops in the world, in addition to maize and rice [1,2]. In Korea, per capita wheat consumption is 34.2 kg per year, which relies on imports for more than 95% of the requirement since the self-sufficiency rate is very low [3]. However, consumer preference for domestic wheat is increasing due to the development of new varieties and an increase in domestic wheat flour-derived food products [4].

Plant viruses are among the pathogens that infect wheat, and more than 50 plant viruses are known to infect wheat worldwide [5,6]. Symptoms of viral infection in wheat include discolored leaves, leaf mosaics, stunted plants, dwarfism, chlorosis, and leaf lesions. Further, yield loss in wheat is caused by reduced stands and decreased root development, water–use efficiency, plant vigor, and nutrient uptake [7,8]. Wheat stripe mosaic virus (WSMV), barley yellow dwarf virus (BYDV), cereal yellow dwarf virus (CYDV), soilborne wheat mosaic virus, and wheat spindle streak mosaic virus are known as economically important plant viruses infecting wheat worldwide [7,9,10,11]. These viruses were reported to reduce wheat yields by an average of 7–10% annually. In addition, WSMV is known to cause significant economic damage by causing up to 100% losses [12,13]. BYDV and CYDV cause yellow dwarf disease, among the most critical plant viruses worldwide. These viruses are transmitted persistently by aphids and are classified into ten different species, belonging to the genera *Luteovirus* (CYDV-RPS, and BYDV-RPV, -RMV), *Polerovirus* (BYDV-PAV, -PAS, -MAV, -kerII, and -ker III) or in the as-yet-unclassified genera (BYDV-GPV and BYDV-SGV) [7,14,15]. Despite the potential damage caused by plant viruses in wheat production, no comprehensive study has been conducted on the incidences and diversity of wheat viruses in Korea.

Various serological and molecular methods are commonly used to detect and diagnose plant viruses. However, since these approaches can only detect known target viruses, it is still a challenge to identify non-target or novel unknown viruses [16,17]. The limitations can be overcome by high-throughput sequencing (HTS) techniques combined with metagenomic analysis. HTS is a very powerful technology to accurately identify viral entities in plant samples, including viruses and viroids in symptomatic as well as asymptomatic plants without any prior knowledge [18,19]. HTS approaches have been utilized for discovery, diagnostic, and evolutionary studies over the past two decades. Several HTS platforms have been launched in the past few years, including second-generation platforms SOLiD, Roche 454, Illumina, and Ion Torrent, and third-generation platforms PacBio and Nanopore with longer reads [20,21,22].

The Illumina sequencing platform has taken a leading position as the most frequently applied HTS technology in plant virology research, including virus detection, discovery, or diversity studies and whole genome sequencing, due to its highest accuracy (>99.9%) and high throughput (from 2 to 750 Gb) [21,23]. Though the “high throughput” is not sufficient for virus diagnosis, high-end computer equipment and appropriate bioinformatics tools are also required to process hundreds of gigabases of massive output data consisting of short reads (from 50 bp to 300 bp) for de novo assembly and sequence alignment [24,25].

On the other hand, MinION, implemented by Oxford Nanopore Technologies (ONT), is a portable single-molecule sequencer designed for laboratories with limited resources and is being used as an efficient tool for plant virus diagnosis and identification [25,26,27]. Nanopore technology enables the sequencing of long-length reads (approximately 300 kb) in real-time by measuring information on voltage changes induced as a nucleic acid strand anchored by a molecular motor protein passes through a biological nanopore [28]. Earlier, the MinION used had an error rate of up to 15%; however, since the base-calling algorithm has been improved, a consensus accuracy of over 99.9% has been reported recently [29,30]. The flongle flow cells (up to 2.8 Gb of data per run) accessed using the flongle adapter to the MinION have relatively low performance, but their cost-effectiveness and portability make them a particularly attractive platform for low-throughput applications [31].

In this study, two methods from the HTS platform and a bioinformatics pipeline were used to detect and identify various plant viruses infecting wheat.

## 2. Results

### 2.1. Virus Identification by ONT Sequencing

The ONT sequencing was performed on libraries from three regions (Jeonbuk; JN, Jeonnam; JB, and Gyeonnam; GN), with 772,355 raw reads in the JN-ONT library, 490,030 raw reads in the JB-ONT library, and 1,177,844 raw reads in the GN-ONT library. The average read length for each library was 412.8 bp (JN-ONT library), 340.1 bp (JB-ONT library), and 325.1 bp (GN-ONT library) on average, with a maximum read length of 8222 bp (JN-ONT library), 12,301 bp (JB-ONT library), and 78,921 bp (GN-ONT library). Adapter trimming and quality filtering (>Q7) were performed on the raw reads, obtaining 761,277, 485,491, and 1,160,201 trimmed reads in the JN-ONT, JB-ONT, and GN-ONT libraries, respectively (Table 1).

**Figure 1:**
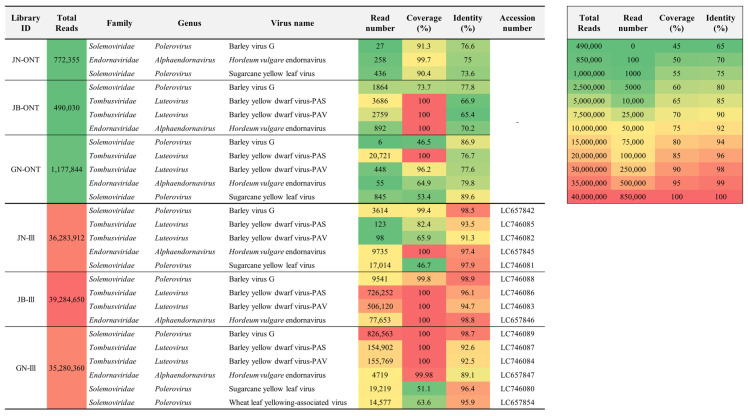
Heatmap comparing total reads, read number, coverage (%), and identity (%) data for ONT and Illumina platforms.—means no data.

Virus identification using the WIMP workflow from the trimmed reads obtained from the three libraries led to the identification of barley virus G (BVG) (27 reads), *Hordeum vulgare* endornavirus (HvEV) (258 reads), and sugarcane yellow leaf virus (ScYLV) (436 reads) from JN-ONT library, BVG (1864 reads), BYDV-PAV (3686 reads), and BYDV-PAS (2759 reads) from JB-ONT library, and BVG (6 reads), BYDV-PAV (20,721 reads), BYDV-PAS (448 reads), HvEV (55 reads), and ScYLV (845 reads) from GN-ONT library (Figure 1).

### 2.2. Virus Identification by Illumina Sequencing

HTS of the three libraries generated 5,478,870,712 bp (JN-Ill library), 5,931,982,150 bp (JB-Ill library), and 5,327,334,360 bp (GN-Ill library) raw paired-end reads. The number of virus-associated reads in the raw reads was 19,138 (JN-Ill library), 220,452 (JB-Ill library), and 120,742 (GN-Ill library). In addition, 21 (JN-Ill library), 38 (JB-Ill library), and 46 (GN-Ill library) virus-annotated contigs (Rank1, e-value ≤ 1 × 10^−5^) were obtained (Table 1).

BLASTN identified plant viruses with contigs from each library. BVG (JN-Ill library; 3614 reads, JB-Ill library; 9541 reads, and GN-Ill library; 826,563 reads), BYDV-PAS (JN-Ill library; 123 reads, JB-Ill library; 726,252 reads, and GN-Ill library; 154,902 reads), BYDV-PAV (JN-Ill library; 98 reads, JB-Ill library; 506,120 reads, and GN-Ill library; 155,769 reads), and HvEV (JN-Ill library; 9735 reads, JB-Ill library; 77,653 reads, and GN-Ill library; 4719 reads) were classified as common from all libraries in three regions. In addition, ScYLV (17,014 reads) from the JN-Ill library and ScYLV (19,219 reads) and WLYaV (14,577 reads) from the GN-Ill library were identified (Figure 1).

### 2.3. Genome Assembly

**Figure 2:**
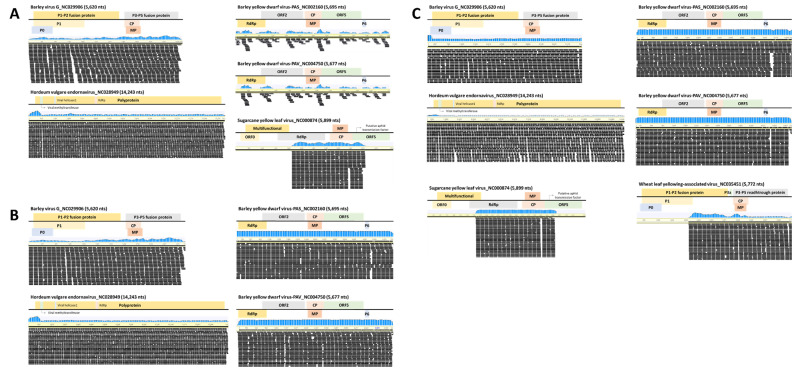
Genome assembly of viral genomes identified by the Illumina platform. Genome mapping of five viruses from JN-Ill library (**A**), four viruses from JB-Ill library (**B**), and six viruses from GN-Ill library (**C**) identified in wheat. The genome structure of the virus and the blue graph is the mapping distribution of the reads. The black bar represents read.

Genome assembly was performed from the reference genomes of all viruses identified by ONT sequencing (Supplementary Figure S1) and Illumina sequencing in wheat. Five viruses identified from the JN-Ill library had coverage of 46.7% (ScYLV) to 100% (HvEV) and identity of 91.3% (BYDV-PAV) to 98.5% (BVG). Four viruses identified from the JB-Ill library had coverage of 99.8% (BVG) to 100% (BYDV-PAS, BYDV-PAV, and HvEV) and identity of 94.7% (BYDV-PAV) to 98.9% (BVG). Next, the six viruses identified from the GN-Ill library had coverage of 51.1% (WLYaV) to 100% (BVG, BYDV-PAS, and BYDV-PAV) and identity of 89.1% (HvEV) to 98.97% (BVG) (Figure 1 and Figure 2). The consensus sequence of the virus coat protein (CP) identified through Illumina sequencing was deposited at the NCBI database to obtain accession numbers (Figure 1).

Gap filling was performed to obtain the complete consensus sequence of CP in BYDV-PAS and BYDV-PAV identified from the JN-Ill library (data not shown).

### 2.4. Phylogenetic Analyses of Identified Viruses

**Figure 3:**
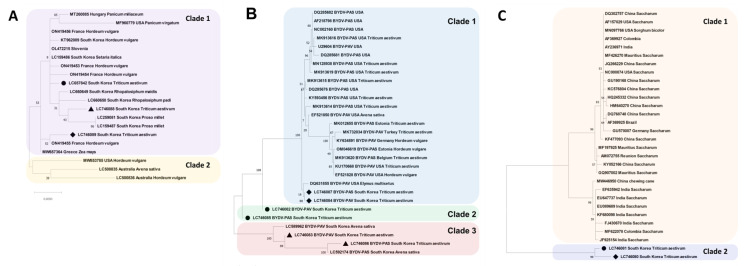
The phylogenetic analysis calculated from the coat protein sequences of barley virus G (BVG) (**A**), barley yellow dwarf virus-PAS (BYDV-PAS) and barley yellow dwarf virus-PAV (BYDV-PAV) (**B**), and sugarcane yellow leaf virus (ScYLV) (**C**). The black circles indicate isolates identified from the JN-Ill library, black triangles from the JB-Ill library, and black diamonds from the GN-Ill library.

Phylogenetic tree analysis was performed on the complete CP consensus sequences of BVG, BYDV-PAS, BYDV-PAV, and ScYLV identified by Illumina sequencing (Figure 3). The maximum likelihood method based on BVG and ScYLV was divided into one larger clade (clade 1) and one smaller clade (clade 2). Further, BYDV-PAS and BYDV-PAV were divided into two larger clades (clades 1 and 2) and one smaller clade (clade 3).

The phylogenetic tree for BVG showed that all three isolates belonged to clade 1. The isolate (LC657842) from the JN-Ill library was close to France isolate (ON419454) from *H. vulgare*, the isolate (LC746088) from JB-Ill library was close to the South Korean isolates (LC259081 and LC159487) from Proso millet, and the isolate (LC746089) from GN-Ill library was closely related to France isolate (ON419455) from *H. vulgare* (Figure 3A).

The phylogenetic tree for BYDV-PAS and BYDV-PAV showed that six isolates belonged to clades 1, 2, and 3, respectively. BYDV-PAS (LC746087) and BYDV-PAV (LC746084) isolates from GN-Ill library belonged to clade 1 and were close to the USA isolate (DQ631855) from *Elymus multisetus*. BYDV-PAS (LC746085) and BYDV-PAV (LC746082) isolates from the JN-Ill library are divided into only two isolates in clade 2. BYDV-PAS isolate (LC746086) from JB-Ill library was close to the South Korean isolate (LC592174) from *Avena sativa*, and BYDV-PAV isolate (LC746083) was also closely related to the other South Korean isolate (LC589962) from *Avena sativa* (Figure 3B).

The phylogenetic tree for ScYLV showed that two isolates (LC746081 and LC746080) from JN-Ill and GN-Ill libraries were distant from other isolates (clade 1) and divided into clade 2 (Figure 3C).

### 2.5. Validation of the Identified Virus by RT-PCR

**Figure 4:**
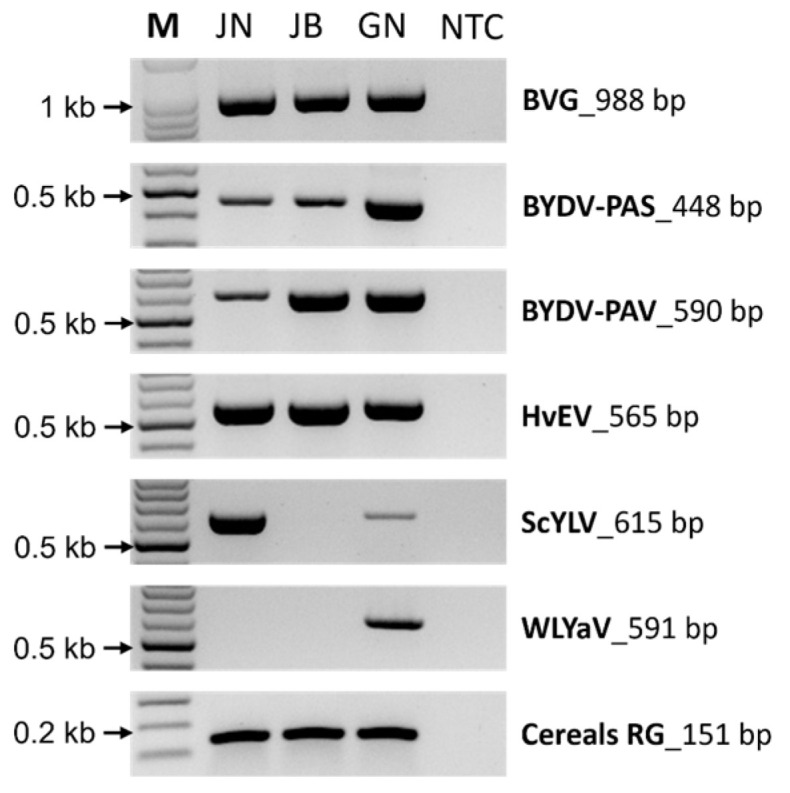
Confirmation of viruses identified by the Illumina platform using RT-PCR. RT-PCR was used to amplify the viruses from wheat samples using specific primer sets to viruses identified using the Illumina platform. The cereals RG was used as an internal control. M, DNA size 100 bp ladder; NTC, no template control.

RT-PCR was performed using gene-specific primer sets to confirm the presence of viruses identified by Illumina sequencing (Figure 4). All viruses identified in the three libraries through Illumina sequencing confirmed the expected amplicon size in RT-PCR, and the cereals reference genome was also amplified.

## 3. Discussion

Wheat (*Triticum aestivum* L.) is widely grown as a crop with high economic value. Wheat viruses continue to pose a major threat to wheat production and global food security [32]. Recently, various HTS platforms have been utilized for detecting undocumented or newly putative viruses and for complete virome analysis in wheat [32,33,34,35,36,37,38]. However, no studies have been reported identifying wheat-infecting viruses in Korea; therefore, we aimed to determine the composition of the wheat virome by utilizing field samples from three major domestic wheat-producing regions through two HTS platforms. The HTS platform is considered one of the most exciting technologies for plant virus diagnostics in integrated disease management programs, as it facilitates the highly sensitive identification and characterization of viruses [39]. Previous studies using HTS revealed the infection of novel viruses in wheat, such as wheat virus Q, wheat yellow stunt-associated betaflexivirus, and the first umbra-like associated RNA viruses named wheat umbra-like virus [32,34,35]. In most studies utilizing the HTS platform, WSMV has been identified as the most critical virus worldwide; however, it was not identified in the current study. This study identified the presence of five viruses, namely BVG, HvEV, BYDV-PAS, BYDV-PAV, ScYLV, and WLYaV, through wheat virome analysis.

In the present study, virus infection was monitored using the Flongle flow cell of the ONT platform for rapid diagnosis of wheat viruses. HTS sequencing with the ONT platform of three different field samples (Jeonnam, Jeonbuk, and Gyeongnam) revealed the identification of three viruses (BVG, HvEV, and ScYLV) from JN-ONT library, four viruses (BVG, BYDV-PAS, BYDV-PAV, and HvEV) from JB-ONT library, and five viruses (BVG, BYDV-PAS, BYDV-PAV, HvEV, and ScYLV) from GN-ONT library. When genome mapping was performed based on the data obtained from the ONT platform, the average coverage was high (84.6%), while the sequence identity was relatively low (76.3%). These results can be attributed to the low sequence data throughput and high error rate (approximately 10%) of the flow cell, which are the biggest drawbacks of the ONT platform to date [28]. Notwithstanding, for users with limited bioinformatics knowledge, the EPI2ME workflow provided by the ONT platform is an attractive tool for plant virus diagnosis. The taxonomic classification was performed using the “What’s in my pot?” application, which is one of the additional options available in EPI2ME. This feature allows for the diagnosis of plant viruses without the need for software through a computer command-line interface [40]. In addition, the new version 10 flow cell provides up to 99.99% base-calling accuracy, and several algorithms can further improve raw read accuracy by upwards of 3% using the Guppy toolkit [31,40]. Therefore, we applied the ONT platform to detect viruses infecting wheat rapidly and showed that it is a promising strategy for wheat virus identification.

As mentioned above, the wheat viruses analyzed by the ONT platform exhibited low sequence identity. We further analyzed wheat viruses using the Illumina platform to verify this finding. Using the Illumina platform, we detected five viruses (BVG, BYDV-PAS, BYDV-PAV, HvEV, and ScYLV) in JN-Ill, four viruses (BVG, BYDV-PAS, BYDV-PAV, and HvEV) in JB-lII, and six viruses (BVG, BYDV-PAS, BYDV-PAV, HvEV, ScYLV, and WLYaV) in GN-Ill, including viruses identified on the ONT platform. The Illumina platform identified more viral species than the ONT platform. This detection difference could be attributed to the higher total read numbers obtained on the Illumina platform (ranging from 490,030 to 1,177,844 reads), as well as the lower base-calling accuracy of the ONT platform (Figure 1).

BVG and HvEV were consistently present in all libraries obtained from the HTS-based platform. BVG was first detected and reported in barley plants that exhibited viral symptoms similar to those caused by BYDV in domestic barley. BVG infection has also been reported in other plant species, including proso millet (*Panicum miliaceum*) and foxtail millet (*Setaria italica*) [41,42,43,44]. HvEV is an endornavirus identified in various organisms, including plants, fungi, and oomycetes. This virus is generally considered non-pathogenic and exhibits a persistent lifestyle. HvEv has recently been reported to infect barley in Korea [45,46,47].

BYDV is transmitted by the bird cherry-oat aphid (*Rhopalosiphum padi*) and is the most devastating cereal crop virus causing ~11 to 33% of yield loss in wheat fields worldwide [48]. BYDV infections have been reported in barley, oats, and wheat in Korea [43,49]. Our study identified infections with the BYDV strains PAS and PAV, while other strains, such as CYDV, were not detected. Interestingly, BYDV-PAS and BYDV-PAV were only identified in the JN-Ill library, not in the JN-ONT library, suggesting that the ONT approach may not be efficient in detecting viruses at low titers (i.e., BYDV-PAS with 123 reads and BYDV-PAV with 98 reads) (Figure 1).

ScYLV is one of the most prevalent viruses causing huge economic losses to sugarcane production worldwide [50]. In the current study, it was identified in JN and GN libraries. ScYLV has been artificially inoculated into wheat (*Triticum* spp.) according to previous studies, but it has never been detected in natural occurrence [50,51]. The ScYLV identified in this study exhibited low coverage and sequence identity and formed a distinct clade from other isolates in the phylogenetic tree analysis (Figure 1 and Figure 3C). The presence of ScYLV was confirmed by RT-PCR (Figure 4). Therefore, this is the first report on the natural infection of ScYLV in wheat.

WLYaV was exclusively identified in the GN-Ill library with 95.39% sequence identity and 63.6% coverage (Figure 1). Previous studies have shown that WLYaV is closely related to ScYLV regarding sequence identity and phylogenetic relationships [52]. Notably, WLYaV was not detected in the GN-ONT library, possibly due to the inability of the ONT platform to differentiate between WLYaV and ScYLV.

In summary, six different plant viruses were identified in wheat samples from three geographically different regions in Korea using the ONT and Illumina platforms. The ONT platform was comparable to the commonly used Illumina platform in viral species detection in wheat samples, though it may not accurately identify viruses with low titers. Despite this limitation, the ONT platform is an attractive tool for efficiently monitoring plant viruses due to its fast running time, portability, and low cost. Although the HTS approach identified ScYLV in wheat for the first time, further studies are required to characterize it.

To prevent the risk of bioinformatics errors or experimental contamination in plant virus diagnosis based on HTS platforms, it is crucial to validate the findings with supplementary methods, such as RT-PCR and pathogenicity, using back inoculation (based on Koch’s postulates). While mechanical inoculation is the typical approach for conducting biological characterization of viruses, it is worth noting that many of the viruses identified in wheat are primarily transmitted by insect vectors, leading to practical limitations. Therefore, additional studies, such as an artificial inoculation system using infectious clones and insect vector-mediated inoculation, are required for biological validation.

These findings provide a preliminary understanding of wheat virus incidences and risk categories in Korea. Knowledge of the wheat virome helps to understand the complex interactions between different viruses and their hosts. This understanding allows for the development of integrated disease management strategies, including the selection of resistant cultivars, deployment of biological control agents, and implementation of cultural practices that limit viral spread. Wheat virome analysis can also help early detection of viral infections, even before visible symptoms appear. This early detection is crucial for implementing rapid response measures, such as quarantine, removal of infected plants, and preventive treatments to minimize the spread of viruses. In addition, wheat virome can provide insights into the transmission mechanisms of plant viruses, including vectors, such as insects and fungi. Understanding the transmission pathways helps in designing targeted control strategies that focus on interrupting the transmission cycle, such as insecticides. Overall, the current study’s outcomes would further help to prepare appropriate control measures.

## 4. Materials and Methods

### 4.1. Sample Preparation and RNA Extraction

**Figure 5:**
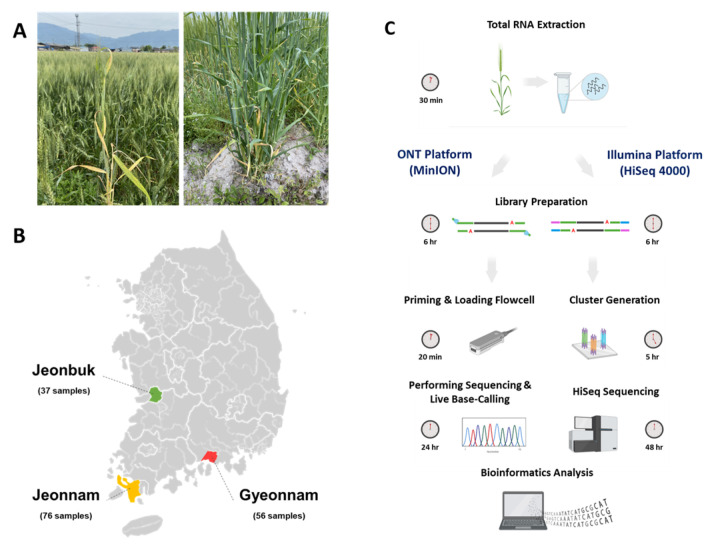
Symptoms such as leaf mosaic, dwarfism, chlorosis, and discolored leaves of wheat (**A**), wheat collection location by region in Korea (**B**), and Workflow of the two HTS platforms (**C**). Created with Biorender (biorender.com (accessed on 2 June 2023)).

Wheat (*Triticum aestivum* L. var. Baekkang) showing yellowing and mosaic symptoms were collected from three regions (Jeonbuk, Jeonnam, and Gyeonnam) in Korea in May 2022 (Figure 5A). Wheat samples were collected and pooled from 37 samples from Jeonbuk, 76 from Jeonnam, and 56 from Gyeonnam (Figure 5B). According to the manufacturer’s instructions, total RNA was extracted from wheat samples pooled for each region using the Beniprep^®^ Super Plant RNA extraction kit (InVirusTech Co., Gwangju, Republic of Korea). The extracted total RNA was stored at −80 °C for subsequent library preparation after measuring RNA quality and concentration using a BioDrop spectrophotometer (Biochrom Ltd., Cambridge, UK).

### 4.2. Library Preparation for ONT Platform and Virus Identification

For library construction (Figure 5C), DNA contamination was removed from total RNA extracted using the RapidOut DNA Removal Kit (Thermo Fisher Scientific, Waltham, MA, USA). QIAseq FastSelect rRNA Plant Kit (Qiagen, Hilden, Germany) was used to remove wheat rRNA (ribodepletion) and purified with AMPure XP beads (Beckman Coulter, Brea, CA, USA). The purified RNA concentration was measured using a Qubit 4 Fluorometer (Thermo Fisher Scientific, Waltham, MA, USA). Libraries were prepared using the Direct cDNA Sequencing Kit (SQK-DCS109; Oxford Nanopore Technologies, Oxford, UK) following the manufacturer’s instructions and previous studies [31,53]. The prepared libraries were loaded on one Flongle Flow Cell (FLO-FLG001, R9.4.1; Oxford Nanopore Technologies, Oxford, UK) per sample, and the sequencing run was started on a MinION device with a Flongle adapter (Oxford Nanopore Technologies, Oxford, UK). Sequencing was performed for 24 h using MinKNOW software (version 21.11.7; Oxford Nanopore Technologies, Oxford, UK) and base-calling in High-accuracy mode with Guppy (version 5.1.12; Oxford Nanopore Technologies, Oxford, UK). Sequencing adapters were removed with Geneious Prime (version 21.1.1). Viruses were identified in ‘What’s in my Pot?’ (WIMP) workflow of EPI2ME Agent (version 3.4.2) and NCBI BLASTn using FASTQ files generated by the ONT platform.

### 4.3. Library Preparation for Illumina Platform and Virus Identification

The total RNA was measured for quality and concentration using the Agilent 2100 bioanalyzer and RNA 6000 pico chip (Agilent Technologies, Palo Alto, CA, USA). Ribosomal RNA was removed using the Ribo-zero rRNA removal kit for plants (Illumina, San Diego, CA, USA) and purified using RNAClean XP beads (Beckman Coulter, Brea, CA, USA). According to the manufacturer’s instructions, libraries were constructed with the TruSeq stranded total RNA library prep kit (Illumina, San Diego, CA, USA). The sequencing was performed on an Illumina Hiseq 4000 (Illumina, San Diego, CA, USA) through a library clustering process to generate 151 bp paired-end reads. The raw reads were trimmed with base quality <30 and minimum length <50 bp using the Trim Galore (version 0.6.4) [54], and virus reads were classified with the Deconseq (version 0.4.3) [55] using the NCBI database. The trimmed reads were assembled using SPAdes assembler (version 3.14.1) [56], and read mapping was performed using BWA-MEM software (0.7.17-r1198-dirty) [57]. The obtained contigs were identified as plant viruses using NCBI BLASTn based on rank1, cut-off E-value of 1 × 10^−5^.

### 4.4. Genome Mapping of Viral Sequences

To confirm genome coverage and identity of plant viruses identified through the HTS platform, reads were mapped using Geneious Prime (version 21.1.1) ‘Map to Reference’ tool with default settings (Medium Sensitivity/Fast).

### 4.5. Phylogenetic Analysis

The phylogenetic analyses were performed using coat protein (CP) sequences of four species (BVG, BYDV-PAS, BYDV-PAV, and ScYLV) with isolates deposited in NCBI GenBank from the plant viruses identified through the Illumina platform. The CP nucleotide sequences were aligned using the BioEdit Sequence Alignment Editor with ClustalW Multiple alignments (version 7.0.5.3). Phylogenetic analysis was performed using the maximum-likelihood method by MEGA X (version 10.2.4) with the nucleotide substitutions selected Tamura-Nei model [58,59]. Bootstrap values were calculated using 1000 replicates.

### 4.6. Confirmation of the Identified Viruses by RT-PCR

The presence of viruses identified by the Illumina platform was confirmed using RT-PCR and Sanger sequencing. RT-PCR was performed with specific primer sets (Supplementary Table S1) and the Suprimescript RT-PCR premix (Genet Bio, Daejeon, Republic of Korea) according to the manufacturer’s instructions. In addition, RT-PCR products were individually cloned using the AccuRapid™ TA Cloning kit (Bioneer, Daejeon, Republic of Korea) and further sequenced and validated using BLASTN search.

### 4.7. Data Availability

The FASTQ files generated in this study have been deposited in the NCBI Sequence Read Archive (SRA) repository under accession numbers SRR22371364 (JN-ONT), SRR22371365 (JB-ONT), SRR22371366 (GN-ONT), SRR16774430 (JN-Ill), SRR16774429 (JB-Ill), and SRR16774428 (GN-Ill) in the NCBI BioProject database as PRJNA776691. Plant virus sequences identified on the Illumina platform have been deposited with individual accession numbers in NCBI GenBank.

## Figures and Tables

**Table 1:** Data summary from ONT and Illumina platforms.

**HTS** **Platform**	**Library** **ID**	**Raw Reads**				**Trimmed Reads**				**SRA** **Accession** **Number**
		**Total** **Reads**	**Mini** **Read** **Length**	**Max** **Read** **Length**	**Mean** **Read** **Length**	**Total** **Reads**	**Mini** **Read** **Length**	**Max** **Read** **Length**	**Mean** **Read** **Length**	
ONT	JN-ONT	772,355	91	8222	412.8	761,277	1	8222	296.3	SRR22371364
	JB-ONT	490,030	55	12,301	340.1	485,491	1	12,301	272	SRR22371365
	GN-ONT	1,177,844	82	78,921	325.1	1,160,201	1	78,921	221.8	SRR22371366
**HTS** **platform**	**Library** **ID**	**Total reads based (bp)**	**Total reads**	**Q30 (%)**	**Virus data**			**Virus annotated contigs**		**SRA** **Accession** **Number**
					Sum reads		Sum bases (bp)			
Illumina	JN-Ill	5,478,870,712	36,283,912	95.52	19,138		2,550,819	21		SRR16774430
	JB-Ill	5,931,982,150	39,284,650	95.2	220,452		32,179,884	38		SRR16774429
	GN-Ill	5,327,334,360	35,280,360	95.04	120,742		17,615,293	46		SRR16774428

## Data Availability

The data presented in this study are available in the GenBank database (www.ncbi.nlm.nih.gov (accessed on 27 November 2021)) under accession numbers listed in the text.

## Supplementary Materials

The following supporting information can be downloaded at: https://www.mdpi.com/article/10.3390/plants12122374/s1, Figure S1: The genome assembly of identified viruses by the ONT platform.; Table S1: Information on primer sets used for RT-PCR. Refs. [60,61,62,63] have been cited in Supplementary Materials.

## References

[1] Yang W., Liu D., Li J., Zhang L., Wei H., Hu X., Zheng Y., He Z., Zou Y. (2009). Synthetic hexaploid wheat and its utilization for wheat genetic improvement in China. J. Genet. Genom..

[2] Weaver G.L. (2001). A miller’s perspective on the impact of health claims. Nutr. Today.

[3] Jo Y.J., You T.Y., Shin T.W., Sung H.J., Lee H.W., Kwak J.E., Kang T.-S., Lee J., Jeong H.S. (2022). Quality characteristics of noodle made from domestic early maturity and high yield wheat cultivars. J. Korean Soc. Food Sci. Nutr..

[4] Choi Y.-S., Lee J.-K., Choi Y.-H., Kim Y.-H., Kang C.-S., Shin M. (2015). Quality characteristics of wheat flours from new released Iksan370 with long spike and domestic wheat cultivars. Korean J. Food Cook. Sci..

[5] Velandia M., Rejesus R.M., Jones D.C., Price J.A., Workneh F., Rush C.M. (2010). Economic impact of Wheat streak mosaic virus in the Texas High Plains. Crop Prot..

[6] Ordon F., Habekuss A., Kastirr U., Rabenstein F., Kühne T. (2009). Virus resistance in cereals: Sources of resistance, genetics and breeding. J. Phytopathol..

[7] Rotenberg D., Bockus W.W., Whitfield A.E., Hervey K., Baker K.D., Ou Z., Laney A.G., De Wolf E.D., Appel J.A. (2016). Occurrence of viruses and associated grain yields of paired symptomatic and nonsymptomatic tillers in Kansas winter wheat fields. Phytopathology.

[8] Kleczewski N., Chapara V., Bradley C.A. (2020). Occurrence of Viruses and *Clavibacter michiganensis* in Winter Wheat in Illinois, 2009 to 2011 and 2019 to 2020. Plant Health Prog..

[9] Singh K., Wegulo S.N., Skoracka A., Kundu J.K. (2018). Wheat streak mosaic virus: A century old virus with rising importance worldwide. Mol. Plant Pathol..

[10] D’arcy C., Burnett P. (1995). Barley Yellow Dwarf: 40 Years of Progress.

[11] Clover G., Ratti C., Henry C. (2001). Molecular characterization and detection of European isolates of Soil-borne wheat mosaic virus. Plant Pathol..

[12] Almas L.K., Price J.A., Workneh F., Rush C.M. (2016). Quantifying economic losses associated with levels of wheat streak mosaic incidence and severity in the Texas High Plains. Crop Prot..

[13] Brakke M. (1987). Virus diseases of wheat. Wheat Wheat Improv..

[14] Pichon E., Souquet M., Armand T., Jacquot E. (2022). Wheat cultivars and natural-based substances: Impacts on epidemiological parameters of yellow dwarf disease. Plant Pathol..

[15] King A.M., Lefkowitz E.J., Mushegian A.R., Adams M.J., Dutilh B.E., Gorbalenya A.E., Harrach B., Harrison R.L., Junglen S., Knowles N.J. (2018). Changes to taxonomy and the International Code of Virus Classification and Nomenclature ratified by the International Committee on Taxonomy of Viruses (2018). Arch. Virol..

[16] Nabi S.U., Baranwal V.K., Rao G.P., Mansoor S., Vladulescu C., Raja W.H., Jan B.L., Alansi S. (2022). High-throughput RNA sequencing of mosaic infected and non-infected apple (*Malus* × *domestica* Borkh.) cultivars: From detection to the reconstruction of whole genome of viruses and viroid. Plants.

[17] Mehetre G.T., Leo V.V., Singh G., Sorokan A., Maksimov I., Yadav M.K., Upadhyaya K., Hashem A., Alsaleh A.N., Dawoud T.M. (2021). Current developments and challenges in plant viral diagnostics: A systematic review. Viruses.

[18] Fajardo T.V.M., Bertocchi A.A., Nickel O. (2020). Determination of the grapevine virome by high-throughput sequencing and grapevine viruses detection in Serra Gaúcha. Brazil. Rev. Ceres.

[19] Kreuze J.F., Perez A., Untiveros M., Quispe D., Fuentes S., Barker I., Simon R. (2009). Complete viral genome sequence and discovery of novel viruses by deep sequencing of small RNAs: A generic method for diagnosis, discovery and sequencing of viruses. Virology.

[20] Chalupowicz L., Dombrovsky A., Gaba V., Luria N., Reuven M., Beerman A., Lachman O., Dror O., Nissan G., Manulis-Sasson S. (2019). Diagnosis of plant diseases using the Nanopore sequencing platform. Plant Pathol..

[21] Dong Z.-X., Lin C.-C., Chen Y.-K., Chou C.-C., Chen T.-C. (2022). Identification of an emerging cucumber virus in Taiwan using Oxford nanopore sequencing technology. Plant Methods.

[22] Šašić Zorić L., Janjušević L., Djisalov M., Knežić T., Vunduk J., Milenković I., Gadjanski I. (2023). Molecular Approaches for Detection of Trichoderma Green Mold Disease in Edible Mushroom Production. Biology.

[23] Goodwin S., McPherson J.D., McCombie W.R. (2016). Coming of age: Ten years of next-generation sequencing technologies. Nat. Rev. Genet..

[24] Barba M., Czosnek H., Hadidi A. (2014). Historical perspective, development and applications of next-generation sequencing in plant virology. Viruses.

[25] Pecman A., Adams I., Gutiérrez-Aguirre I., Fox A., Boonham N., Ravnikar M., Kutnjak D. (2022). Systematic Comparison of Nanopore and Illumina Sequencing for the Detection of Plant Viruses and Viroids Using Total RNA Sequencing Approach. Front. Microbiol..

[26] Boykin L.M., Sseruwagi P., Alicai T., Ateka E., Mohammed I.U., Stanton J.-A.L., Kayuki C., Mark D., Fute T., Erasto J. (2019). Tree lab: Portable genomics for early detection of plant viruses and pests in sub-saharan africa. Genes.

[27] Bronzato Badial A., Sherman D., Stone A., Gopakumar A., Wilson V., Schneider W., King J. (2018). Nanopore sequencing as a surveillance tool for plant pathogens in plant and insect tissues. Plant Dis..

[28] Lu H., Giordano F., Ning Z. (2016). Oxford Nanopore MinION sequencing and genome assembly. Genom. Proteom. Bioinform..

[29] Chang J.J.M., Ip Y.C.A., Bauman A.G., Huang D. (2020). MinION-in-ARMS: Nanopore sequencing to expedite barcoding of specimen-rich macrofaunal samples from autonomous reef monitoring structures. Front. Mar. Sci..

[30] Van Dijk E.L., Jaszczyszyn Y., Naquin D., Thermes C. (2018). The third revolution in sequencing technology. Trends Genet..

[31] Liefting L.W., Waite D.W., Thompson J.R. (2021). Application of Oxford Nanopore technology to plant virus detection. Viruses.

[32] Redila C.D., Prakash V., Nouri S. (2021). Metagenomics analysis of the wheat virome identifies novel plant and fungal-associated viral sequences. Viruses.

[33] Singh K., Jarošová J., Fousek J., Huan C., Kundu J.K. (2020). Virome identification in wheat in the Czech Republic using small RNA deep sequencing. J. Integr. Agric..

[34] Kondo H., Yoshida N., Fujita M., Maruyama K., Hyodo K., Hisano H., Tamada T., Andika I.B., Suzuki N. (2021). Identification of a Novel Quinvirus in the Family *Betaflexiviridae* That Infects Winter Wheat. Front. Microbiol..

[35] Fu S., Zhang T., He M., Sun B., Zhou X., Wu J. (2021). Molecular characterization of a novel wheat-infecting virus of the family *Betaflexiviridae*. Arch. Virol..

[36] Ranabhat N.B., Fellers J.P., Bruce M.A., Rupp J.L.S. (2023). Brome mosaic virus detected in Kansas wheat co-infected with other common wheat viruses. Front. Plant Sci..

[37] Hodge B., Paul P., Stewart L.R. (2020). Occurrence and high-throughput sequencing of viruses in Ohio wheat. Plant Dis..

[38] Fellers J.P., Webb C., Fellers M.C., Shoup Rupp J., De Wolf E. (2019). Wheat virus identification within infected tissue using nanopore sequencing technology. Plant Dis..

[39] Gallo Y., Marín M., Gutiérrez P. (2021). Detection of RNA viruses in *Solanum quitoense* by high-throughput sequencing (HTS) using total and double stranded RNA inputs. Physiol. Mol. Plant Pathol..

[40] Petersen L.M., Martin I.W., Moschetti W.E., Kershaw C.M., Tsongalis G.J. (2019). Third-generation sequencing in the clinical laboratory: Exploring the advantages and challenges of nanopore sequencing. J. Clin. Microbiol..

[41] Park C., Oh J., Min H.-G., Lee H.-K., Lee S.-H. (2017). First report of barley virus g in proso millet (*Panicum miliaceum*) in Korea. Plant Dis..

[42] Oh J., Park C., Min H.-G., Lee H.-K., Yeom Y.-A., Yoon Y., Lee S.-H. (2017). First report of barley virus g in foxtail millet (*Setaria italica*) in Korea. Plant Dis..

[43] Jo Y., Bae J.-Y., Kim S.-M., Choi H., Lee B.C., Cho W.K. (2018). Barley RNA viromes in six different geographical regions in Korea. Sci. Rep..

[44] Zhao F., Lim S., Yoo R.H., Igori D., Kim S.-M., Kwak D.Y., Kim S.L., Lee B.C., Moon J.S. (2016). The complete genomic sequence of a tentative new polerovirus identified in barley in South Korea. Arch. Virol..

[45] Candresse T., Marais A., Sorrentino R., Faure C., Theil S., Cadot V., Rolland M., Villemot J., Rabenstein F. (2016). Complete genomic sequence of barley (*Hordeum vulgare*) endornavirus (HvEV) determined by next-generation sequencing. Arch. Virol..

[46] Fukuhara T., Gibbs M. (2012). Family *endornaviridae*. Virus Taxonomy: Ninth Report of the International Committee on Taxonomy of Viruses.

[47] Roossinck M.J. (2010). Lifestyles of plant viruses. Philos. Trans. R. Soc. B Biol. Sci..

[48] Walls J., Rajotte E., Rosa C. (2019). The past, present, and future of barley yellow dwarf management. Agriculture.

[49] Kim N.-K., Lee H.-J., Kim S.-M., Jeong R.-D. (2022). Identification of viruses infecting oats in Korea by metatranscriptomics. Plants.

[50] ElSayed A.I., Komor E., Boulila M., Viswanathan R., Odero D.C. (2015). Biology and management of sugarcane yellow leaf virus: An historical overview. Arch. Virol..

[51] Bouallegue M., Mezghani-Khemakhem M., Makni H., Makni M. (2014). First report of Sugarcane yellow leaf virus infecting barley in Tunisia. Plant Dis..

[52] Zhang P., Liu Y., Liu W., Cao M., Massart S., Wang X. (2017). Identification, characterization and full-length sequence analysis of a novel polerovirus associated with wheat leaf yellowing disease. Front. Microbiol..

[53] Lee H.-J., Cho I.-S., Jeong R.-D. (2022). Nanopore Metagenomics Sequencing for Rapid Diagnosis and Characterization of Lily Viruses. Plant Pathol. J..

[54] Krueger F. (2015). Trim Galore. A Wrapper Tool Around Cutadapt and FastQC to Consistently Apply Quality and Adapter Trimming to FastQ Files.

[55] Schmieder R., Edwards R. (2011). Fast identification and removal of sequence contamination from genomic and metagenomic datasets. PLoS ONE.

[56] Bankevich A., Nurk S., Antipov D., Gurevich A.A., Dvorkin M., Kulikov A.S., Lesin V.M., Nikolenko S.I., Pham S., Prjibelski A.D. (2012). SPAdes: A new genome assembly algorithm and its applications to single-cell sequencing. J. Comput. Biol..

[57] Li H., Durbin R. (2009). Fast and accurate short read alignment with Burrows–Wheeler transform. Bioinformatics.

[58] Kumar S., Stecher G., Li M., Knyaz C., Tamura K. (2018). MEGA X: Molecular evolutionary genetics analysis across computing platforms. Mol. Biol. Evol..

[59] Tamura K., Nei M., Kumar S. (2004). Prospects for inferring very large phylogenies by using the neighbor-joining method. Proc. Natl. Acad. Sci. USA.

[60] Laney A.G., Acosta-Leal R., Rotenberg D. (2018). Optimized yellow dwarf virus multiplex PCR assay reveals a common occurrence of Barley yellow dwarf virus-PAS in Kansas winter wheat. Plant Health Prog..

[61] Malmstrom C.M., Shu R. (2004). Multiplexed RT-PCR for streamlined detection and separation of barley and cereal yellow dwarf viruses. J. Virol. Methods.

[62] Viswanathan R., Karuppaiah R., Balamuralikrishnan M. (2010). Detection of three major RNA viruses infecting sugarcane by multiplex reverse transcription-polymerase chain reaction (multiplex-RT-PCR). Australas. Plant Pathol..

[63] Balaji B., Bucholtz D.B., Anderson J.M. (2003). Barley yellow dwarf virus and Cereal yellow dwarf virus quantification by real-time polymerase chain reaction in resistant and susceptible plants. Phytopathology.

