# Supplementary material for: Analysis of Wheat Virome in Korea Using Illumina and Oxford Nanopore Sequencing Platforms
Source: Plants (Basel). 2023 Jun 19;12(12):2374. doi: 10.3390/plants12122374 (PMC10303500; doi:10.3390/plants12122374)
Supplement: Supplementary file 1 [file plants-12-02374-s001.zip › Fig. S1.pdf]

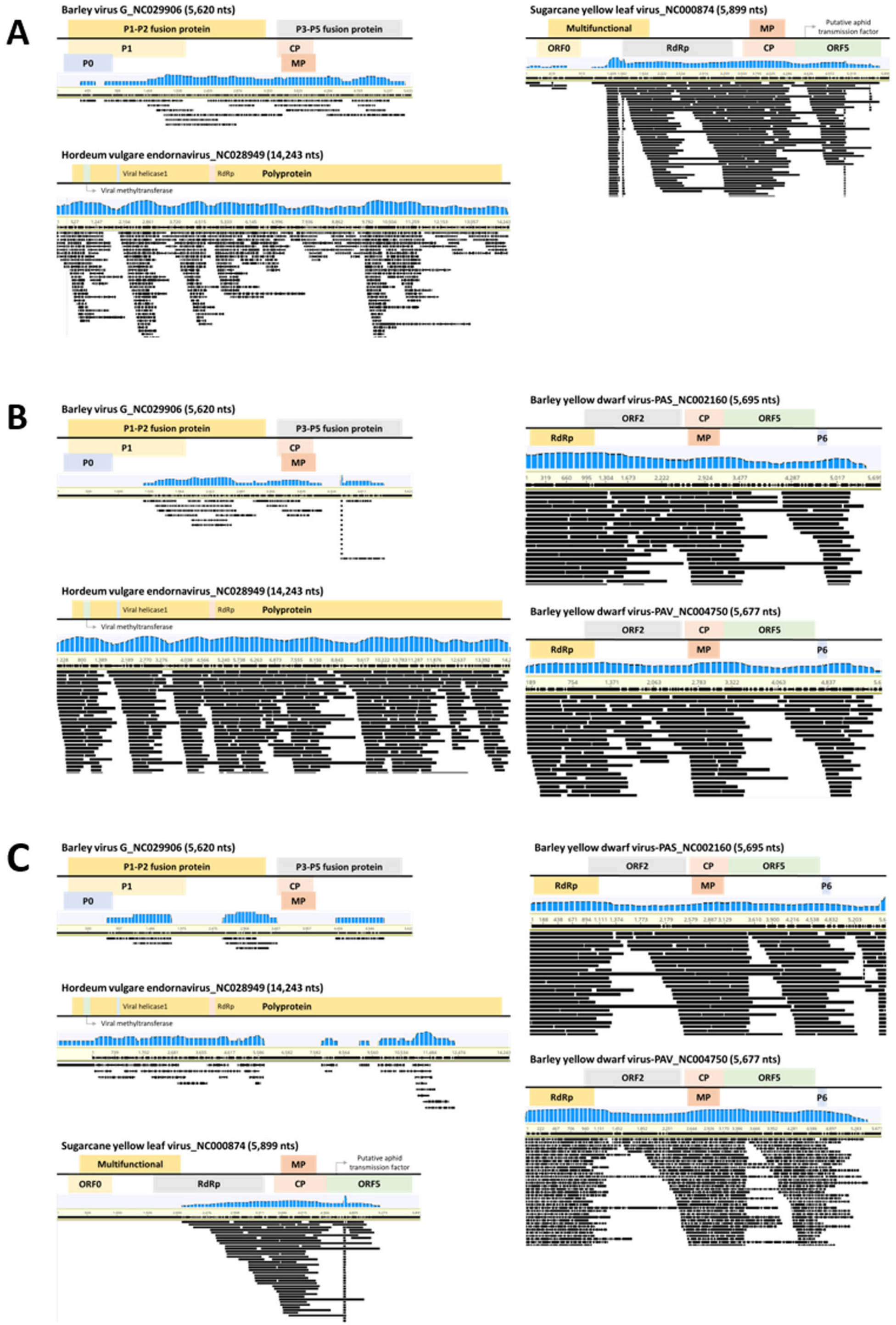
A
Barley virus G_NC029906 (5,620 nts)
P1-P2 fusion protein
P3-P5 fusion protein
P1
CP
P0
MP
Sugarcane yellow leaf virus_NC000874 (5,899 nts)
Multifunctional
MP
Putative aphid transmission factor
ORF0
RdRp
CP
ORF5
Hordeum vulgare endornavirus_NC028949 (14,243 nts)
Viral helicase1
RdRp
Polyprotein
Viral methyltransferase
B
Barley virus G_NC029906 (5,620 nts)
P1-P2 fusion protein
P3-P5 fusion protein
P1
CP
P0
MP
Barley yellow dwarf virus-PAS_NC002160 (5,695 nts)
ORF2
CP
ORF5
RdRp
MP
P6
Hordeum vulgare endornavirus_NC028949 (14,243 nts)
Viral helicase1
RdRp
Polyprotein
Viral methyltransferase
Barley yellow dwarf virus-PAV_NC004750 (5,677 nts)
ORF2
CP
ORF5
RdRp
MP
P6
C
Barley virus G_NC029906 (5,620 nts)
P1-P2 fusion protein
P3-P5 fusion protein
P1
CP
P0
MP
Barley yellow dwarf virus-PAS_NC002160 (5,695 nts)
ORF2
CP
ORF5
RdRp
MP
P6
Hordeum vulgare endornavirus_NC028949 (14,243 nts)
Viral helicase1
RdRp
Polyprotein
Viral methyltransferase
Barley yellow dwarf virus-PAV_NC004750 (5,677 nts)
ORF2
CP
ORF5
RdRp
MP
P6
Sugarcane yellow leaf virus_NC000874 (5,899 nts)
Multifunctional
MP
Putative aphid transmission factor
ORF0
RdRp
CP
ORF5
