# Supplementary material for: Analysis of Wheat Virome in Korea Using Illumina and Oxford Nanopore Sequencing Platforms
Source: Plants (Basel). 2023 Jun 19;12(12):2374. doi: 10.3390/plants12122374 (PMC10303500; doi:10.3390/plants12122374)
Supplement: Supplementary file 1 [file plants-12-02374-s001.zip › plants-2424722-supplementary.pdf]

Supplementary Table S1. Information on primer sets used for RT-PCR.

Target virus	Primer name	Sequence 5'-3'	Product size (bp)	Reference
BVG	Fwd<br>Rev	GTGAGTTGCAAGTACTGGAT<br>GTACCCTGCCGAAAGTGTT	988	[1]
BYDV-PAS	Fwd<br>Rev	GGAGACGACTGTGTCATCATCACTGAG<br>TGTCGTTTGTGATAGGTGTCTCC	448	[2]
BYDV-PAV	Fwd<br>Yan-R	ACCTAGACGCGCAAATCAAA<br>TGTTGAGGAGTCTACCTATTTG	590	[3]
HvEV	10992F1<br>11556R1	CCCAGAGTAGACAAAGTGATGGAAAG<br>CTCCTGGTTTCAATTTGTCGCTG	565	[4]
ScYLV	F615<br>R615	GGATCCATGAATACGGGCGCTAACCGYYCAC<br>AGATCTGTGTTGGGGRAGCGTCGCYTACC	615	[5]
WLYaV	Fwd<br>Rev	ATGAATACGGGAGGTAAC<br>CTATTTTGGATTCTGGAATTGA	591	This study
Cereals RG	18S rRNA-F<br>18S rRNA-R	GTGACGGGTGACGGAGAATT<br>GACACTAATGCGCCCGGTAT	151	[6]

References

1. Park, C.; Oh, J.; Min, H.-G.; Lee, H.-K.; Lee, S.-H. First report of barley virus g in proso millet (*Panicum miliaceum*) in korea. *Plant Dis.* **2017**, *101*, 393-393. https://doi.org/10.1094/PDIS-07-16-0952-PDN
2. Laney, A.G.; Acosta-Leal, R.; Rotenberg, D. Optimized yellow dwarf virus multiplex PCR assay reveals a common occurrence of Barley yellow dwarf virus-PAS in Kansas winter wheat. *Plant Health Prog.* **2018**, *19*, 37-43. https://doi.org/10.1094/PHP-09-17-0056-RS
3. Malmstrom, C.M.; Shu, R. Multiplexed RT-PCR for streamlined detection and separation of barley and cereal yellow dwarf viruses. *J. Virol. Methods* **2004**, *120*, 69-78. https://doi.org/10.1016/j.jviromet.2004.04.005
4. Jo, Y.; Bae, J.-Y.; Kim, S.-M.; Choi, H.; Lee, B.C.; Cho, W.K. Barley RNA viromes in six different geographical regions in Korea. *Sci. Rep.* **2018**, *8*, 13237. https://doi.org/10.1038/s41598-018-31671-4
5. Viswanathan, R.; Karuppaiah, R.; Balamuralikrishnan, M. Detection of three major RNA viruses infecting sugarcane by multiplex reverse transcription-polymerase chain reaction (multiplex-RT-PCR). *Australas. Plant Pathol.* **2010**, *39*, 79-84. https://doi.org/10.1071/AP09059
6. Balaji, B.; Bucholtz, D.B.; Anderson, J.M. Barley yellow dwarf virus and Cereal yellow dwarf virus quantification by real-time polymerase chain reaction in resistant and susceptible plants. *Phytopathology* **2003**, *93*, 1386-1392. https://doi.org/10.1094/PHYTO.2003.93.11.1386
